# γ-Cyclodextrin Co-Ingestion Enhances the Bioavailability of Perilla Oil, Regardless of Inclusion Complex Formation

**DOI:** 10.3390/ijms26167776

**Published:** 2025-08-12

**Authors:** Keisuke Yoshikiyo, Hidehisa Shimizu, Hitomi Okada, Atsunori Hasegawa, Tatsuyuki Yamamoto

**Affiliations:** 1 Institute of Agricultural and Life Sciences, Academic Assembly, Shimane University, 1060 Nishikawatsu-Cho, Matsue 690-8504, Shimane, Japan; tyamamot@life.shimane-u.ac.jp; 2 Graduate School of Natural Science and Technology, Shimane University, 1060 Nishikawatsu-Cho, Matsue 690-8504, Shimane, Japan; 3 Faculty of Life and Environmental Sciences, Shimane University, 1060 Nishikawatsu-Cho, Matsue 690-8504, Shimane, Japan; 4 The United Graduate School of Agricultural Sciences, Tottori University, 4-101 Koyama-Minami, Tottori 680-8553, Tottori, Japan; 5 Interdisciplinary Center for Science Research, Shimane University, 1060 Nishikawatsu-Cho, Matsue 690-8504, Shimane, Japan

**Keywords:** γ-cyclodextrin, perilla oil, α-linolenic acid, bioavailability, inclusion complex, omega-3 fatty acids, lipid absorption, functional food, dietary supplement, rat model

## Abstract

Perilla oil, a plant-derived lipid rich in α-linolenic acid (ALA), has demonstrated enhanced bioavailability when administered as an inclusion complex with γ-cyclodextrin (γ-CD). Crucially, it remains unclear whether this enhancement requires complex formation or can be achieved simply by co-ingestion. To address this, we compared the effects of a γ-CD–perilla oil inclusion complex to the effects of a physical mixture of the two on the plasma fatty acid profiles of rats fed these preparations for four weeks. Both treatment groups showed significant alterations in plasma fatty acid composition compared to the control group. Notably, our results indicated no significant differences between the inclusion complex and physical mixture groups. These findings suggest that γ-CD facilitates the intestinal absorption of perilla oil through co-ingestion, irrespective of its complexation status. This highlights the potential of γ-CD as a practical and effective delivery aid for improving the bioavailability of ALA-rich oils. Further studies are warranted to elucidate the underlying mechanisms and their applicability to human nutrition.

## 1. Introduction

Perilla oil, derived from the seeds of *Perilla frutescens* widely cultivated in East Asia, is a plant-based lipid source particularly rich in α-linolenic acid (ALA) [1]. ALA, a polyunsaturated omega-3 fatty acid, is well regarded for its extensive health-promoting properties [2,3]. Numerous studies have linked dietary ALA intake to a reduced risk of cardiovascular diseases [4,5,6], improved cognitive performance [6,7], neuroprotection and liver damage amelioration [8,9], and significant anti-inflammatory effects [10]. Perilla oil stands out as one of the most concentrated natural sources of ALA, making it a valuable nutritional component for individuals aiming to increase their omega-3 fatty acid intake [6,11].

Despite the well-documented and scientifically validated health benefits associated with ALA and other omega-3 fatty acids, the actual bioavailability of perilla oil—defined as the proportion of an ingested nutrient that is absorbed and becomes available for physiological use in the body—remains suboptimal. This challenge is common for oils rich in ALA and similar omega-3 fatty acids [2]. The limited bioavailability primarily arises from inefficient gastrointestinal absorption and metabolic utilization in vivo, as reported in both animal and human studies [2,12,13,14]. However, the process that facilitates lipid absorption is lipolysis, defined as the enzymatic hydrolysis of triglycerides (TG) [12,13]. Unfortunately, polyunsaturated fatty acids (PUFAs) such as ALA exhibit lower rates of this process than saturated and monounsaturated fatty acids [14,15]. This reduced lipolytic efficiency impairs their incorporation into mixed micelles, which are essential for the efficient intestinal absorption of dietary lipids, consequently reducing ALA uptake across the intestinal epithelium and diminishing its functional efficacy.

To address these absorption challenges and maximize the physiological effects of omega-3 fatty acids as dietary supplements, various industrial and formulation strategies have been developed [16,17]. These include the use of free fatty acid forms, chemical re-esterification, and advanced delivery systems such as microencapsulation and oil-in-water (O/W) emulsification [17,18]. These approaches aim to enhance the solubility and dispersion of omega-3 fatty acids in the gastrointestinal environment, thereby improving their bioavailability. Given the rapidly expanding global nutraceutical and functional food market for omega-3 fatty acids, there is a growing demand for simpler, cost-effective, and broadly applicable methods to reliably improve their intestinal absorption and systemic bioefficacy [16,17].

Cyclodextrins (CDs), cyclic oligosaccharides composed of six (α-CD), seven (β-CD), or eight (γ-CD) α-D-glucopyranose units linked by α-1,4-glycosidic bonds, are characterized by their unique toroidal structure with a hydrophilic exterior and hydrophobic central cavity [19]. This architecture enables the formation of non-covalent host–guest inclusion complexes with a wide range of lipophilic or poorly water-soluble molecules [20,21]. Consequently, CDs are widely utilized in pharmaceutical and food sciences to improve the aqueous solubility, chemical stability, and bioavailability of various bioactive compounds, including plant-derived oils, fat-soluble vitamins, and polyphenols [19,22,23,24,25,26].

Building on this concept, our previous studies have demonstrated that forming an inclusion complex between perilla oil and γ-CD can significantly enhance the bioavailability of ALA, perilla oil’s primary omega-3 fatty acid [27,28]. This enhancement was evidenced by increased plasma ALA concentrations following oral administration of the complex in animal studies [28]. These findings suggest that γ-CD-based inclusion complexation may be a practical and efficient strategy to overcome the absorption limitations of PUFA-rich oils and improve their nutritional impact. However, it remains unclear whether the observed improvements in bioavailability truly necessitate the molecular encapsulation of perilla oil within the γ-CD cavity or if simple physical co-administration of perilla oil and γ-CD—without forming a true inclusion complex—is sufficient to produce comparable physiological effects. Therefore, the present study was designed to address this specific question. To this end, we conducted a comparative analysis of plasma fatty acid profiles in rats fed either a preformed inclusion complex of perilla oil and γ-CD or a physical mixture of the two, assessing the differences in absorption and metabolic utilization. The inclusion complex between γ-CD and perilla oil was prepared through non-covalent interactions, such as hydrophobic interactions and van der Waals forces, without any covalent modification of the ALA moiety present in perilla oil.

## 2. Results

### 2.1. Food Intake, Weight Gain, and Blood Biochemical Parameters

During the four-week feeding period, no significant differences were observed in body weight gain between the experimental groups. Overall food intake was largely comparable, although a statistically significant reduction was noted in the group which received a diet containing an inclusion complex of perilla oil and γ-CD (IC group) compared with the control (CTRL) group (*p* = 0.0443, Table 1). In contrast, the group which received a diet comprising a physical mixture of perilla oil and γ-CD (PM group) showed no significant difference in food intake compared to the CTRL (*p* = 0.5855) or IC (*p* = 0.7591) groups.

Table 2 shows the blood biochemical parameters after the four-week feeding period. No significant differences were observed in the plasma levels of aminotransferase (AST) or alanine aminotransferase (ALT) between the IC and PM groups, and both were comparable to those of the CTRL group, indicating no apparent hepatotoxicity. Blood concentrations of total cholesterol (T-CHO) and TG, which are markers of lipid metabolism, also showed no significant differences among the groups. The T-CHO levels were comparable across all groups. TG levels exhibited high interindividual variability, regardless of dietary treatment. Neither the IC nor PM group showed significant differences in TG concentrations compared to the CTRL group. Glucose (GLU) concentrations were similar among all groups.

### 2.2. Plasma Fatty Acid Composition

**Figure 1:**
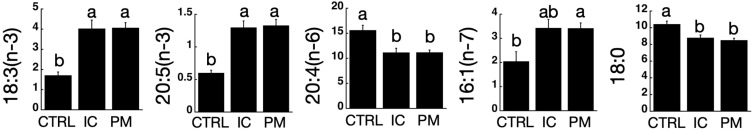
Relative composition (%) of selected plasma fatty acids in rats after four weeks of dietary intervention. The five fatty acids shown exhibited statistically significant differences among groups: α-linolenic acid (ALA, 18:3(n-3)), eicosapentaenoic acid (EPA, 20:5(n-3)), arachidonic acid (ARA, 20:4(n-6)), palmitoleic acid (16:1(n-7)), and stearic acid (C18:0). Data are presented as mean ± standard error (SE) (*n* = 10 per group). Different letters above the bars indicate significant differences among groups, as determined by the Kruskal–Wallis test followed by Dunn’s post hoc test (*p* < 0.05). CTRL—control group fed the AIN-93G diet; IC—group fed the diet containing an inclusion complex of perilla oil and γ-cyclodextrin; PM—group fed the diet containing a physical mixture of perilla oil and γ-cyclodextrin.

Table 3 summarizes the relative composition of 11 plasma fatty acids, each accounting for approximately 0.5% or more of the total fatty acids. Compared to the CTRL group, both the IC and PM groups, which were fed diets containing 87.4% soybean oil and 12.6% perilla oil, exhibited notable alterations in the composition of several fatty acids. Figure 1 shows significant differences in plasma fatty acid profiles among the experimental groups. Notably, the IC and PM groups exhibited highly similar patterns in their fatty acid compositions. This similarity was evident despite the distinct methods used to prepare the two formulations—namely, preformed molecular inclusion versus simple physical mixing. These findings suggest that co-ingestion of perilla oil and γ-CD, regardless of complex formation, leads to comparable metabolic outcomes.

The proportion of ALA, the principal component of perilla oil, was significantly elevated in both the IC and PM groups compared to the CTRL group. Similarly, the level of eicosapentaenoic acid (EPA), a metabolic derivative of ALA, was significantly increased. In contrast, docosahexaenoic acid (DHA), a terminal omega-3 fatty acid, showed no significant changes. Arachidonic acid (ARA) levels were decreased in the IC and PM groups compared to those in the CTRL group. Palmitoleic acid, an endogenously synthesized monounsaturated fatty acid, was significantly higher in the IC and PM groups than in the CTRL group. Among the saturated fatty acids, stearic acid levels were significantly reduced in the IC and PM groups, whereas the levels of other saturated fatty acids remained unchanged. No statistically significant differences were observed among the groups for the remaining fatty acids analyzed. Notably, all five fatty acids that differed significantly from those in the CTRL group showed nearly identical patterns in the IC and PM groups, suggesting that γ-CD promotes the intestinal availability of perilla oil components, regardless of complex formation.

## 3. Discussion

### 3.1. Comparable Enhancement in the Bioavailability of Perilla Oil Through Co-Ingestion with γ-Cyclodextrin

During the four-week feeding period, food intake, body weight gain, and blood biochemical parameters remained consistent between the IC and PM groups, indicating stable physiological conditions and enabling a valid comparison of their plasma fatty acid profiles to assess the impact of γ-CD on perilla oil bioavailability. A slight but significant reduction in food intake was observed in the IC group compared to the CTRL group (*p* = 0.0443); however, this did not translate into a significant difference in body weight gain or other systemic markers, suggesting a minimal physiological impact on the overall study results. This could potentially be attributed to γ-CD and other soluble dietary fibers, which contribute to a greater sense of satiety and influence food intake [29,30,31,32]. However, further investigation is required to confirm the specific effect of γ-CD.

Among the 11 plasma fatty acids analyzed, five showed statistically significant alterations in the IC and PM groups compared to the CTRL group. Importantly, the compositional changes were nearly identical between the IC and PM groups. The most notable changes were observed in ALA, the major component of perilla oil, and its downstream metabolite EPA, both of which were significantly elevated in the IC and PM groups. These increases likely reflect the direct contribution of dietary ALA and its metabolic conversion to EPA.

Concomitantly, a reduction in ARA, a representative omega-6 fatty acid, was also observed. This may be attributable to the competitive relationship between omega-3 and omega-6 fatty acids, which share common metabolic enzymes involved in desaturation and elongation [33,34]. This reduced ARA may offer advantages in modulating inflammatory responses, thereby contributing to chronic disease prevention. Therefore, the increase in omega-3 fatty acids may suppress omega-6 pathways, a phenomenon supported by previous studies [35,36]. This shift in fatty acid composition, notably the increased EPA and decreased ARA levels, may not only reflect altered substrate availability but also indicate favorable modulation of inflammatory pathways through lipid mediator production.

Changes in endogenous fatty acid levels were also observed: palmitoleic acid, synthesized via stearoyl-CoA desaturase-1 (SCD-1), was significantly increased, whereas stearic acid levels were significantly decreased in both the IC and PM groups. These alterations may reflect shifts in hepatic lipid metabolism driven by dietary polyunsaturated fatty acids (PUFAs) such as ALA, which suppress lipogenic gene expression and promote β-oxidation [37,38,39] and contribute to alterations of PUFA profiles in metabolic syndrome [40,41]. Further investigation is required to determine the exact mechanisms underlying these changes in composition.

Overall, the similar fatty acid profiles in the IC and PM groups strongly suggest that γ-CD facilitates the intestinal absorption of perilla oil, regardless of whether a true inclusion complex is formed. This finding implies that the co-ingestion of γ-CD with perilla oil is sufficient to improve its bioavailability, thus providing a practical alternative to pre-complexation in functional food development.

### 3.2. Potential Mechanisms of γ-Cyclodextrin-Facilitated Absorption

The potential mechanisms by which γ-CD may enhance lipid absorption are multifaceted. γ-CD may stabilize lipid emulsions, thereby increasing the surface area available for lipase activity [22]. Furthermore, it may enhance substrate accessibility by associating with lipid–water interfaces [27].

For dietary TGs to be absorbed, they must first undergo emulsification by bile acids, followed by enzymatic hydrolysis by lipases and incorporation of the resulting free fatty acids and monoacylglycerols into mixed micelles [13,42,43]. The enhanced absorption of ALA and EPA observed in both the IC and PM groups suggests that γ-CD may support one or more of these processes.

A crucial aspect to consider is the potential interactions between γ-CD and endogenous emulsifiers in the body. Certain CDs, notably β-CD [44,45], exhibit a strong affinity for bile acids, potentially interfering with micelle formation and thereby impairing intestinal lipid absorption. Similarly, α-CD can disrupt micellar structure through interactions with phospholipids [46]. In contrast, γ-CD demonstrates a lower binding affinity for bile acids and lecithin, which likely minimizes its interference with emulsification [22,47,48]. This understanding is further corroborated by our recent study [47], which provides additional evidence supporting the notion that γ-CD does not adversely affect lipid absorption, even in the context of elevated gastrointestinal 12-hydroxylated bile acid levels. Notably, concentrations of 12-hydroxylated bile acids in the gastrointestinal tract are known to be particularly elevated in conditions such as high-fat diet and obesity [49]. Unlike α-CD and β-CD, which can disrupt micelle formation by binding strongly to bile acids or phospholipids, γ-CD exhibits a much lower affinity for these endogenous emulsifiers. This particular property is presumed to underlie its minimal interference with the micellar solubilization of lipids. Furthermore, our findings suggest that γ-CD may contribute to the maintenance of bile acid homeostasis without impairing lipid digestion or absorption, highlighting its potential safety and functional advantages as a dietary additive. This distinct interaction profile likely accounts for the absence of adverse effects on lipid absorption observed in the IC and PM groups in the present study. While these hypotheses are supported by previous studies and the observed enhancement in ALA and EPA absorption, it is important to note that no direct microscopic imaging, in vivo lipase activity assays, or intestinal micelle analyses were conducted in the present study. Therefore, the proposed mechanisms should be interpreted as speculative, and further experimental validation will be necessary in future research.

Although γ-CD can form inclusion complexes with TGs present in perilla oil, these complexes are sparingly soluble in water [50]. Their recoverability by filtration from aqueous dispersions suggests that they primarily exist as water-insoluble aggregates. In the gastrointestinal (GI) tract, such complexes may serve as reservoirs that gradually release oil into bile salt/phospholipid emulsions, thereby enhancing emulsification and subsequent absorption. However, in vitro complexation requires several hours of stirring, which is longer than the typical small intestinal transit time. This implies that the formation of inclusion complexes between perilla oil TGs and γ-CD is unlikely to occur rapidly in the GI tract, particularly in the PM group. Therefore, while preformed inclusion complexes can serve as slow-release reservoirs [21], their in vivo contribution to the enhancement of lipid absorption is presumed to be limited in this study [24].

An in vitro lipolysis assay using porcine pancreatic lipase demonstrated that γ-CD accelerated the release of free fatty acids from perilla oil TGs, particularly ALA [27,28]. Saturated fatty acids, such as palmitic and stearic acids, were hydrolyzed more rapidly than unsaturated fatty acids, including oleic, linoleic, and ALA. The presence of γ-CD facilitated ALA release, suggesting that γ-CD may increase substrate accessibility or alter the emulsion stability. This could occur because γ-CD interacts at the lipid–water interface, potentially modifying the interfacial tension or lipid droplet size, thereby improving lipase adsorption and catalytic efficiency [51]. This observation aligns with the findings of Akanbi et al., who reported that flaxseed oil, rich in ALA, showed a lower extent of lipolysis than oils rich in monounsaturated fatty acids in an in vitro digestion model, which they attributed to the lower catalytic efficiency of pancreatic lipase toward PUFAs, highlighting the inherent difficulty in digesting PUFA-rich TGs [15]. Although this supports a mechanistic role for γ-CD in lipid digestion, further studies are required to confirm this effect in vivo.

Collectively, these findings suggest that γ-CD enhances the bioavailability of perilla oil primarily by promoting emulsification and lipolysis without interfering with endogenous emulsification systems. γ-CD is recognized as a Generally Recognized As Safe (GRAS) substance by the U.S. Food and Drug Administration (FDA) and approved as a food additive in several countries, its use as a delivery aid in functional foods is both practical and regulatory feasible [19]. Therefore, co-ingestion with γ-CD may represent a promising and scalable strategy for improving the nutritional efficacy of ALA-rich plant oils, such as perilla oil.

### 3.3. Limitations of the Study and Future Perspectives

While this study provides valuable insights into the mechanism by which γ-CD enhances the bioavailability of perilla oil, it is important to acknowledge certain limitations. First, this study was conducted using a rat model. While invaluable for mechanistic studies, findings from animal models may not always be directly extrapolated to human physiology due to inherent species-specific differences in digestive systems, metabolic rates, and genetic backgrounds [52,53], particularly concerning the unique composition and metabolic activities of the gut microbiota in each species [54], which can significantly influence nutrient absorption and utilization. Therefore, future studies on human subjects are necessary to confirm these findings. Furthermore, although our discussion proposes potential mechanisms for the action of γ-CD, such as enhanced emulsification and lipolysis, direct in vivo evidence demonstrating these specific molecular events within the gastrointestinal tract is still lacking. Future studies should explore more advanced methodologies for studying lipid absorption mechanisms in vivo, such as stable isotope tracing with mass spectrometry, advanced imaging modalities, or novel in vivo sampling techniques, to provide deeper insights into the real-time kinetics and localization of lipid digestion and absorption. Further mechanistic studies are needed to elucidate the precise role of γ-CD in lipid digestion and absorption, such as by evaluating changes in intestinal lipase activity or micelle formation in vivo. Finally, although the observed significant decrease in food intake in the IC group did not result in a significant difference in body weight gain, we acknowledge that even subtle variations in nutrient intake could potentially influence metabolic outcomes. Future experimental designs might benefit from pair feeding or other methods to precisely control dietary intake across groups to eliminate this minor confounding factor.

In terms of future perspectives, this study suggests a practical approach for using γ-CD to enhance ALA bioavailability. It will be crucial to consider the applicability of these findings to various dosage forms (e.g., supplements, functional beverages, and food additives such as dairy products) in future human studies. Furthermore, the effects of minor components in perilla oil other than ALA (e.g., plant sterols and tocopherols) on bioavailability and their interaction with γ-CD are worth investigating. Additionally, comprehensively evaluating the impact of interactions with other dietary nutrients, such as antioxidants, on the stability and absorption of polyunsaturated fatty acids is important for functional food development.

## 4. Materials and Methods

### 4.1. Reagents

γ-CD was purchased from CycloChem Co., Ltd. (Kobe, Japan), and cold-pressed perilla oil was obtained from Okuizumo Nakamura Farm Co., Ltd. (Okuizumo, Japan). Plasma-free and esterified fatty acids were derivatized to their respective methyl esters using a Fatty Acid Methylation Kit (Cat. No. 13246-84, Nacalai Tesque Inc., Kyoto, Japan), according to the manufacturer’s instructions. All other reagents were of analytical grade and purchased from the Fujifilm Wako Pure Chemical Corporation (Osaka, Japan). The inclusion complex of perilla oil and γ-CD was prepared according to a previously described method with minor modifications. Briefly, 30 g of perilla oil was added to an aqueous solution of 170 g γ-CD in 500 mL of distilled water. The mixture was stirred at room temperature (~25 °C) and 600 rpm for 3 h until it turned from transparent to white, indicating complex formation. The disappearance of the uncomplexed oil was confirmed visually, and the mixture was freeze-dried using a DFR-5N-A freeze-dryer (ULVAC Technologies, Methuen, MA, USA). The stability of the preformed inclusion complex between γ-CD and perilla oil was confirmed in a previous study [28], in which the complex maintained its physical and chemical integrity for over 6 days when stored at 40 °C, or up to 3 days at 60 °C under ambient air conditions. Furthermore, the fatty acid profiles of the experimental diets remained unchanged after the feeding period, indicating no degradation of the complex during the experiment.

### 4.2. Animals

All animal experiments were approved by the Animal Care and Use Committee of Shimane University (approval number: MA30-14). Animal care and handling were conducted in accordance with institutional guidelines and the Act on Welfare and Management of Animals (Act No. 105), as well as related regulations in Japan. Male Wistar King A Hokkaido rats (WKAH/HkmSlc; Japan SLC, Kyoto, Japan) were used in this study. The animals were housed individually in plastic cages under controlled environmental conditions (temperature: 26 ± 1 °C; relative humidity: 55 ± 5%; light/dark cycle: 12/12 h). Each rat was treated and monitored independently and was therefore considered an experimental unit for all analyses. Each experimental group consisted of ten rats (*n* = 10). The rats had free access to standard chow and water throughout the study. Body weight and food intake were recorded every day during the feeding period. At the end of the experimental period, the animals were anesthetized with isoflurane (5% for induction and 2% for maintenance) and blood was collected from the abdominal aorta using syringes preloaded with heparin (final concentration: 50 U/mL) and aprotinin (final concentration: 500 kIU/mL). Plasma was separated by centrifugation at 2000× *g* for 10 min at 4 °C and stored at −80 °C until further analysis. The researchers were aware of the group allocations during the experiment and data analysis. Blinding was not performed because of logistical constraints.

### 4.3. Experimental Diets

The experimental diets were formulated based on AIN-93G composition (Table 4). Prior to the study, all rats were acclimated to a standard AIN-93G diet for five days. For the IC diet, 150 g of cellulose and 27.6 g of soybean oil per kg of diet were replaced with 176.5 g of a freeze-dried perilla oil/γ-CD inclusion complex, which contained 150 g of γ-CD and 26.5 g of perilla oil. For the PM diet, 150 g of γ-CD and 27.6 g of perilla oil were added to the diet by replacing the same quantities of cellulose and soybean oil. To prevent unintentional complex formation, perilla oil was first mixed with soybean oil before combining it with the remaining components. Cellulose was used as a control carbohydrate in place of γ-CD to ensure dietary equivalence across experimental groups. While digestible carbohydrates such as maltodextrin may share similar hydrolysis profiles, their use could introduce interpretive ambiguity. In contrast, cellulose is a non-digestible, physiologically inert fiber, allowing for a more rigorous comparison of the effects attributable specifically to γ-CD.

### 4.4. Plasma Fatty Acid Analysis

The fatty acid composition was determined using a gas chromatography–mass spectrometry (GC–MS) system (GCMS-QP2010 Ultra, Shimadzu, Kyoto, Japan) equipped with a DB-5ms capillary column (30 m × 0.25 mm i.d., 0.25 µm film thickness; Agilent Technologies, Santa Clara, CA, USA). Helium was used as the carrier gas at a constant flow rate of 0.89 mL/min. The oven temperature was initially held at 40 °C for 2 min and then ramped to 320 °C at a rate of 6 °C/min. The injector and interface temperatures were set to 280 °C. Mass spectra were acquired in electron ionization (EI) mode at 70 eV. Fatty acid methyl esters (FAMEs) were identified by comparing their retention times and mass spectra with those of the authentic standards.

### 4.5. Plasma Biochemical Analysis

Plasma levels of AST (IU/L), ALT (IU/L), T-CHO (mg/dL), TG (mg/dL), and GLU (mg/dL) were measured using commercially available assay kits with a Hitachi 7180 automatic analyzer (Hitachi Ltd., Tokyo, Japan). All measurements were conducted at the Nagahama Institute of Biochemical Science, Oriental Yeast Co., Ltd. (Shiga, Japan).

### 4.6. Statistical Analysis

Statistical analyses were performed using R software (version 4.4.2; R Foundation for Statistical Computing, Vienna, Austria). Group differences were assessed using the Kruskal–Wallis test followed by Dunn’s post hoc test for pairwise comparisons. Statistical significance was set at *p* < 0.05. Data are presented as mean ± standard error (SE). The Kruskal–Wallis test, a non-parametric method, was employed due to the nature of the data, as it does not assume normal distribution.

## 5. Conclusions

This study demonstrated that the co-ingestion of perilla oil with γ-CD significantly enhanced the bioavailability of ALA and its downstream metabolite EPA in rats. Importantly, this enhancement was observed regardless of whether perilla oil was administered as a preformed inclusion complex or as a simple physical mixture, with comparable increases in plasma omega-3 fatty acid levels across both groups. These findings suggest that the presence of γ-CD during digestion, rather than prior complex formation, is sufficient to promote the intestinal absorption of perilla oil-derived fatty acids in rats. Consequently, labor-intensive complexation steps may not be essential for achieving the functional benefits of γ-CD. This highlights the potential of γ-CD co-ingestion as a simpler and more practical strategy for developing functional foods and supplements aimed at improving omega-3 fatty acid delivery. Such an approach could significantly contribute to public health by providing a more accessible and cost-effective means of enhancing ALA intake, thereby supporting chronic disease prevention and improving overall well-being.

## Figures and Tables

**Table 1:** Food intake and weight gain of rats after four weeks of dietary intervention.

	CTRL	IC	PM
Food intake (g)	457 ± 6 ^a^	430 ± 8 ^b^	443 ± 10 ^ab^
Weight gain (g)	140 ± 4	135 ± 4	135 ± 4

This table delineates the mean food intake (g) and weight gain (g) for the CTRL, IC, and PM groups, along with their respective standard errors. Values are presented as mean ± standard error (*n* = 10 per group). Means within a row that bear different superscript letters (a, b) exhibit significant differences (*p* < 0.05), as determined by the Kruskal–Wallis test followed by Dunn’s post hoc analysis. The CTRL group was administered the AIN-93G diet, the IC group received a diet containing an inclusion complex of perilla oil and γ-cyclodextrin, and the PM group received a diet comprising a physical mixture of perilla oil and γ-cyclodextrin.

**Table 2:** Blood biochemical parameters of rats after four weeks of dietary intervention.

	CTRL	IC	PM
AST (IU/L)	54.7 ± 2.6	52.8 ± 1.3	55.2 ± 2.3
ALT (IU/L)	30.3 ± 2.3	29.1 ± 1.0	30.1 ± 1.6
T-CHO (mg/100 mL)	56.5 ± 1.7	57.4 ± 1.2	56.6 ± 2.0
TG (mg/100 mL)	121 ± 16	166 ± 23	157 ± 17
GLU (mg/100 mL)	189 ± 5	190 ± 2	192 ± 4

This table delineates the mean plasma levels of AST, ALT, T-CHO, TG, and GLU for the CTRL, IC, and PM groups, along with their respective standard errors. The values are presented as mean ± standard error, with a sample size of *n* = 10 per group for each group. The CTRL group was fed the AIN-93G diet, the IC group was fed a diet containing an inclusion complex of perilla oil and γ-cyclodextrin, and the PM group was fed a diet containing a physical mixture of perilla oil and γ-cyclodextrin.

**Table 3:** Relative plasma fatty acid composition (%) in rats after four weeks of dietary intervention with control diet (CTRL), inclusion complex (IC), or physical mixture (PM) of Perilla oil and γ-cyclodextrin.

Fatty Acids	CTRL	IC	PM
	Composition (%)		
14:0	0.71 ± 0.04	0.78 ± 0.04	0.81 ± 0.04
16:0	25.9 ± 0.3	25.0 ± 0.4	25.3 ± 0.2
18:0	10.4 ± 0.4 ^a^	8.8 ± 0.3 ^b^	8.5 ± 0.2 ^b^
16:1(n-7)	2.05 ± 0.40 ^b^	3.43 ± 0.36 ^ab^	3.42 ± 0.22 ^a^
18:1(n-9)	13.4 ± 0.5	14.6 ± 0.5	14.7 ± 0.4
18:2(n-6)	26.1 ± 0.7	27.4 ± 1.0	27.0 ± 0.7
20:3(n-6)	0.55 ± 0.06	0.68 ± 0.06	0.64 ± 0.04
20:4(n-6)	15.6 ± 1.0 ^a^	11.2 ± 0.8 ^b^	11.2 ± 0.5 ^b^
18:3(n-3)	1.71 ± 0.16 ^b^	4.03 ± 0.42 ^a^	4.07 ± 0.26 ^a^
20:5(n-3)	0.60 ± 0.04 ^b^	1.30 ± 0.10 ^a^	1.33 ± 0.09 ^a^
22:6(n-3)	2.06 ± 0.13	2.04 ± 0.12	2.09 ± 0.06

This table presents the percentage composition of various fatty acids (14:0, 16:0, 18:0, 16:1(n-7), 18:1(n-9), 18:2(n-6), 20:3(n-6), 20:4(n-6), 18:3(n-3), 20:5(n-3), 22:6(n-3)) in the plasma of rats across the CTRL, IC, and PM groups, along with their standard errors. Values are expressed as mean ± standard error (*n* = 10 per group). Means within a row that bear different superscript letters (a, b) are significantly different (*p* < 0.05), as determined by the Kruskal–Wallis test followed by Dunn’s post hoc analysis. The CTRL group was fed the AIN-93G diet, the IC group received a diet containing an inclusion complex of perilla oil and γ-cyclodextrin, and the PM group received a diet containing a physical mixture of perilla oil and γ-cyclodextrin.

**Table 4:** Composition of experimental diets based on AIN-93G formulation (g per 3000 g diet).

Ingredients	AIN-93G	IC Diet	PM Diet
	Amount (g)		
Corn Starch	1192.5	1192.5	1192.5
Casein	600	600	600
Maltodextrin	396	396	396
Sucrose	300	300	300
Soybean Oil	210	183.5	183.5
Perilla Oil	—	—	26.5
Cellulose	150	—	—
γ-CD	—	—	150
Inclusion Complexes	—	176.5 *	—
Mineral Mix	105	105	105
Vitamin Mix	30	30	30
L-Cystine	9	9	9
Choline Bitartrate	7.5	7.5	7.5

* The inclusion complex contained 26.5 g of perilla oil and 150 g of γ-cyclodextrin (γ-CD). The CTRL group received the standard AIN-93G diet. IC: inclusion complex of perilla oil and γ-CD; PM: physical mixture of perilla oil and γ-CD.

## Data Availability

The data presented in this study are available on request from the corresponding author.

## References

[1] Guan L., Zhu L., Zhang X., Han Y., Wang K., Ji N., Yao X., Zhou Y., Li B., Chen Q. (2024). Perilla Seed Oil and Protein: Composition, Health Benefits, and Potential Applications in Functional Foods. Molecules.

[2] Lane K.E., Wilson M., Hellon T.G., Davies I.G. (2022). Bioavailability and conversion of plant based sources of omega-3 fatty acids—A scoping review to update supplementation options for vegetarians and vegans. Crit. Rev. Food Sci. Nutr..

[3] Li M., Jiang N., Guo G., Lu S., Li Z., Mu Y., Xia X., Xu Z., Hu Y., Xiang X. (2024). Perilla Seed Oil: A Review of Health Effects, Encapsulation Strategies and Applications in Food. Foods.

[4] Chen H., Leng X., Liu S., Zeng Z., Huang F., Huang R., Zou Y., Xu Y. (2023). Association between dietary intake of omega-3 poly-unsaturated fatty acids and all-cause and cardiovascular mortality among hypertensive adults: Results from NHANES 1999–2018. Clin. Nutr..

[5] Naghshi S., Aune D., Beyene J., Mobarak S., Asadi M., Sadeghi O. (2021). Dietary intake and biomarkers of alpha linolenic acid and risk of all cause, cardiovascular, and cancer mortality: Systematic review and dose-response meta-analysis of cohort studies. BMJ.

[6] Sala-Vila A., Fleming J., Kris-Etherton P.M., Ros E. (2022). Impact of α-Linolenic Acid, the Vegetable ω-3 Fatty Acid, on Cardiovascular Disease and Cognition. Adv. Nutr..

[7] Hashimoto M., Matsuzaki K., Maruyama K., Hossain S., Sumiyoshi E., Wakatsuki H., Kato S., Ohno M., Tanabe Y., Kuroda Y. (2022). Perilla seed oil in combination with nobiletin-rich ponkan powder enhances cognitive function in healthy elderly Japanese individuals: A possible supplement for brain health in the elderly. Food Funct..

[8] Zhang X., Bao J., Zhang Y., Wang X. (2024). Alpha-Linolenic Acid Ameliorates Cognitive Impairment and Liver Damage Caused by Obesity. Diabetes Metab. Syndr. Obes..

[9] Jordão Candido C., Silva Figueiredo P., Del Ciampo Silva R., Candeloro Portugal L., Augusto dos Santos Jaques J., Alves de Almeida J., de Barros Penteado B., Albuquerque Dias D., Marcelino G., Pott A. (2019). Protective Effect of α-Linolenic Acid on Non-Alcoholic Hepatic Steatosis and Interleukin-6 and -10 in Wistar Rats. Nutrients.

[10] Pauls S.D., Rodway L.A., Winter T., Taylor C.G., Zahradka P., Aukema H.M. (2018). Anti-inflammatory effects of α-linolenic acid in M1-like macrophages are associated with enhanced production of oxylipins from alpha-linolenic and linoleic acid. J. Nutr. Biochem..

[11] Yuan Q., Xie F., Huang W., Hu M., Yan Q., Chen Z., Zheng Y., Liu L. (2022). The review of alpha-linolenic acid: Sources, metabolism, and pharmacology. Phytother. Res..

[12] Wang T.Y., Liu M., Portincasa P., Wang D.Q. (2013). New insights into the molecular mechanism of intestinal fatty acid absorption. Eur. J. Clin. Investig..

[13] Duan H., Song W., Zhao J., Yan W. (2023). Polyunsaturated Fatty Acids (PUFAs): Sources, Digestion, Absorption, Application and Their Potential Adjunctive Effects on Visual Fatigue. Nutrients.

[14] Carlier H., Bernard A., Caselli C. (1991). Digestion and absorption of polyunsaturated fatty acids. Reprod. Nutr. Dev..

[15] Akanbi T.O., Sinclair A.J., Barrow C.J. (2014). Pancreatic lipase selectively hydrolyses DPA over EPA and DHA due to location of double bonds in the fatty acid rather than regioselectivity. Food Chem..

[16] Oliver L., Dietrich T., Marañón I., Villarán M.C., Barrio R.J. (2020). Producing Omega-3 Polyunsaturated Fatty Acids: A Review of Sustainable Sources and Future Trends for the EPA and DHA Market. Resources.

[17] Venugopalan V.K., Gopakumar L.R., Kumaran A.K., Chatterjee N.S., Soman V., Peeralil S., Mathew S., McClements D.J., Nagarajarao R.C. (2021). Encapsulation and Protection of Omega-3-Rich Fish Oils Using Food-Grade Delivery Systems. Foods.

[18] Couëdelo L., Lennon S., Abrous H., Chamekh I., Bouju C., Griffon H., Vaysse C., Larvol L., Breton G. (2024). In Vivo Absorption and Lymphatic Bioavailability of Docosahexaenoic Acid from Microalgal Oil According to Its Physical and Chemical Form of Vectorization. Nutrients.

[19] Astray G., Gonzalez-Barreiro C., Mejuto J.C., Rial-Otero R., Simal-Gándara J. (2009). A review on the use of cyclodextrins in foods. Food Hydrocoll..

[20] Szejtli J. (1998). Introduction and General Overview of Cyclodextrin Chemistry. Chem. Rev..

[21] Loftsson T., Brewster M.E. (1996). Pharmaceutical applications of cyclodextrins. 1. Drug solubilization and stabilization. J. Pharm. Sci..

[22] Durante M., Milano F., Caroli M., Giotta L., Piro G., Mita G., Frigione M., Lenucci M.S. (2020). Tomato Oil Encapsulation by α-, β-, and γ-Cyclodextrins: A Comparative Study on the Formation of Supramolecular Structures, Antioxidant Activity, and Carotenoid Stability. Foods.

[23] Davis M.E., Brewster M.E. (2004). Cyclodextrin-based pharmaceutics: Past, present and future. Nat. Rev. Drug Discov..

[24] Martín Del Valle E.M. (2004). Cyclodextrins and their uses: A review. Process Biochem..

[25] Szente L., Szejtli J. (2004). Cyclodextrins as food ingredients. Trends Food Sci. Technol..

[26] Marques H.M.C. (2010). A review on cyclodextrin encapsulation of essential oils and volatiles. Flavour Fragr. J..

[27] Yoshikiyo K., Takahashi M., Narumiya Y., Honda M., Iwasaki K., Ishigaki M., Nagato E.G., Noothalapati H., Shimizu H., Murota K. (2023). Co-ingestion with γ-cyclodextrin improves bioavailability of α-linolenic acid in Perilla frutescens seed oil. Food Hydrocoll. Health.

[28] Yoshikiyo K., Yoshioka Y., Narumiya Y., Oe S., Kawahara H., Kurata K., Shimizu H., Yamamoto T. (2019). Thermal stability and bioavailability of inclusion complexes of perilla oil with γ-cyclodextrin. Food Chem..

[29] Dikeman C.L., Fahey G.C. (2006). Viscosity as related to dietary fiber: A review. Crit. Rev. Food Sci. Nutr..

[30] Slavin J.L., Green H. (2007). Dietary fibre and satiety. Nutr. Bull..

[31] Clark M.J., Slavin J.L. (2013). The effect of fiber on satiety and food intake: A systematic review. J. Am. Coll. Nutr..

[32] So D., Whelan K., Rossi M., Morrison M., Holtmann G., Kelly J.T., Shanahan E.R., Staudacher H.M., Campbell K.L. (2018). Dietary fiber intervention on gut microbiota composition in healthy adults: A systematic review and meta-analysis. Am. J. Clin. Nutr..

[33] Zhang J.Y., Kothapalli K.S., Brenna J.T. (2016). Desaturase and elongase-limiting endogenous long-chain polyunsaturated fatty acid biosynthesis. Curr. Opin. Clin. Nutr. Metab. Care.

[34] Sprecher H. (2000). Metabolism of highly unsaturated *n*-3 and *n*-6 fatty acids. Biochim. Biophys. Acta.

[35] Calder P.C. (2006). n-3 polyunsaturated fatty acids, inflammation, and inflammatory diseases. Am. J. Clin. Nutr..

[36] Simopoulos A.P. (2008). The importance of the omega-6/omega-3 fatty acid ratio in cardiovascular disease and other chronic diseases. Exp. Biol. Med..

[37] Scorletti E., Byrne C.D. (2013). Omega-3 fatty acids, hepatic lipid metabolism, and nonalcoholic fatty liver disease. Annu. Rev. Nutr..

[38] Jump D.B. (2008). N-3 polyunsaturated fatty acid regulation of hepatic gene transcription. Curr. Opin. Lipidol..

[39] Scorletti E., Byrne C.D. (2018). Omega-3 fatty acids and non-alcoholic fatty liver disease: Evidence of efficacy and mechanism of action. Mol. Asp. Med..

[40] Jang H., Park K. (2020). Omega-3 and omega-6 polyunsaturated fatty acids and metabolic syndrome: A systematic review and meta-analysis. Clin. Nutr..

[41] Takić M., Ranković S., Girek Z., Pavlović S., Jovanović P., Jovanović V., Šarac I. (2024). Current Insights into the Effects of Dietary α-Linolenic Acid Focusing on Alterations of Polyunsaturated Fatty Acid Profiles in Metabolic Syndrome. Int. J. Mol. Sci..

[42] Yu L., Bharadwaj S., Brown J.M., Ma Y., Du W., Davis M.A., Michaely P., Liu P., Willingham M.C., Rudel L.L. (2006). Cholesterol-regulated translocation of NPC1L1 to the cell surface facilitates free cholesterol uptake. J. Biol. Chem..

[43] Wang D.Q. (2007). Regulation of intestinal cholesterol absorption. Annu. Rev. Physiol..

[44] Tan X., Lindenbaum S. (1991). Studies on complexation between β-cyclodextrin and bile salts. Int. J. Pharm..

[45] Tepavčević V., Farkaš Agatić Z., Pilipović A., Puača G., Poša M. (2025). Effect of β-Cyclodextrin on the Aggregation Behavior of Sodium Deoxycholate and Sodium Cholate in Aqueous Solution. Molecules.

[46] Furune T., Ikuta N., Ishida Y., Okamoto H., Nakata D., Terao K., Sakamoto N. (2014). A study on the inhibitory mechanism for cholesterol absorption by α-cyclodextrin administration. Beilstein J. Org. Chem..

[47] Yoshikiyo K., Shimizu H., Nagato E.G., Ishizuka S., Yamamoto T. (2025). Comparative Analysis of γ-Cyclodextrin, Perilla Oil, and Their Inclusion Complexes on Liver Injury and Dyslipidemia Associated with Elevated Gastrointestinal 12-Hydroxylated Bile Acid Levels. Molecules.

[48] Wüpper S., Lüersen K., Rimbach G. (2021). Cyclodextrins, Natural Compounds, and Plant Bioactives—A Nutritional Perspective. Biomolecules.

[49] Hori S., Abe T., Lee D.G., Fukiya S., Yokota A., Aso N., Shirouchi B., Sato M., Ishizuka S. (2020). Association between 12α-hydroxylated bile acids and hepatic steatosis in rats fed a high-fat diet. J. Nutr. Biochem..

[50] Dima Ş., Dima C., Iordăchescu G. (2015). Encapsulation of Functional Lipophilic Food and Drug Biocomponents. Food Eng. Rev..

[51] Wang P., Ke Z., Yi J., Liu X., Hao L., Kang Q., Lu J. (2019). Effects of β-cyclodextrin on the enzymatic hydrolysis of hemp seed oil by lipase *Candida sp.*99–125. Ind. Crops Prod..

[52] Baker D.H. (2008). Animal models in nutrition research. J. Nutr..

[53] Gallaher D.D. (1992). Animal models in human nutrition research. Nutr. Clin. Pract..

[54] Li C., Zhang X. (2022). Current in Vitro and Animal Models for Understanding Foods: Human Gut—Microbiota Interactions. J. Agric. Food Chem..

